# Camera Trapping to Assess Status and Composition of Mammal Communities in a Biodiversity Hotspot in Myanmar

**DOI:** 10.3390/ani11030880

**Published:** 2021-03-19

**Authors:** Giacomo Cremonesi, Francesco Bisi, Lorenzo Gaffi, Thet Zaw, Hla Naing, Kyaw Moe, Zarni Aung, Maria V. Mazzamuto, Alessandra Gagliardi, Lucas A. Wauters, Damiano G. Preatoni, Adriano Martinoli

**Affiliations:** 1 Environment Analysis and Management Unit—Guido Tosi Research Group—Department of Theoretical and Applied Sciences, University of Insubria, Via J. H. Dunant, 3, 21100 Varese, Italy; francesco.bisi@gmail.com (F.B.); alessandra.gagliardi@uninsubria.it (A.G.); lucas.wauters@uninsubria.it (L.A.W.); damiano.preatoni@uninsubria.it (D.G.P.); adriano.martinoli@uninsubria.it (A.M.); 2 Istituto Oikos Onlus, Via Crescenzago 1, 20134 Milano, Italy; lnz.gaffi@gmail.com (L.G.); thetzaw.ll90@gmail.com (T.Z.); 3 Wildlife Conservation Society—Myanmar Program No. 12(B-21,22) Narnattaw Road, Shwe Kainnayi Housing, Kamayut Township, Yangon 11041, Myanmar; hnaing@wcs.org (H.N.); phonemyat.moe@gmail.com (K.M.); zarni.wcs@gmail.com (Z.A.); mariavmazzamuto@gmail.com (M.V.M.); 4 School of Natural Resources and Environment, University of Arizona, 1064 E. Lowell Street, Tucson, AZ 85721, USA

**Keywords:** camera trapping, human disturbance, mammal community, occupancy, species richness

## Abstract

**Simple Summary:**

Tropical forests are one of the most impacted habitats in the world due mostly to anthropogenic pressures. Mammal communities are threatened by many human activities but most of the time knowledge of the status of wildlife populations is lacking. In this study, we investigated two mammal communities, in the poorly studied country of Myanmar, characterized by similar environmental conditions but different levels of human pressure and habitat degradation. We found that the disturbed area hosted a community with a lower mammal diversity (species richness) but not altered in its functional composition (trophic niches and body mass) except for the lack of apex predators. There were also differences in the probability of occurrence of two species (Northern red muntjak and clouded leopard) with significantly lower values in the degraded area. The former being the target of hunting for bushmeat consumption and the latter vulnerable and threatened by human activities. These results increase our knowledge on the direct and indirect effects of human disturbance in tropical forest areas in Myanmar and give us important tools for future conservation actions.

**Abstract:**

Tropical forests comprise a critically impacted habitat, and it is known that altered forests host a lower diversity of mammal communities. In this study, we investigated the mammal communities of two areas in Myanmar with similar environmental conditions but with great differences in habitat degradation and human disturbance. The main goal was to understand the status and composition of these communities in an understudied area like Myanmar at a broad scale. Using camera trap data from a three-year-long campaign and hierarchical occupancy models with a Bayesian formulation, we evaluated the biodiversity level (species richness) and different ecosystem functions (diet and body mass), as well as the occupancy values of single species as a proxy for population density. We found a lower mammal diversity in the disturbed area, with a significantly lower number of carnivores and herbivores species. Interestingly, the area did not show alteration in its functional composition. Almost all the specific roles in the community were present except for apex predators, thus suggesting that the effects of human disturbance are mainly effecting the communities highest levels. Furthermore, two species showed significantly lower occupancies in the disturbed area during all the monitoring campaigns: one with a strong pressure for bushmeat consumption and a vulnerable carnivore threatened by illegal wildlife trade.

## 1. Introduction

Tropical forests represent one of the richest ecosystems in the world, hosting biodiversity hotspots and rare species [1]. However, the disruption and degradation rates of this habitat are the highest in the world, mainly due to human activities [2]. Practices such as deforestation and illegal hunting can affect habitats and wildlife communities, changing their diversity and structure with drastic consequences on entire ecosystems [3].

Mammal communities play specific roles in many ecosystems, supporting key functions as consumers, seed dispersers, or predators and prey [4,5]. In addition, several mammals are umbrella species for the whole community [6]. Since deforestation rates have grown rapidly in recent years, it has become important to increase our knowledge on the status of mammalian communities in these altered tropical forest to better preserve them [7]. One of the most evident results is that degraded or altered forests host a lower diversity in mammal communities [3,8,9,10]. In recent years, many studies have focused on species richness and persistence/colonization rates to understand diversity patterns in different communities [11,12,13] while also considering some functional traits like trophic niches (e.g., diet) and body mass size as indicators of the status of the community [14]. These indicators could reveal how animals respond to changes (e.g., body mass) or which role a species has in ecosystem functions (e.g., trophic niches) [15], thus allowing for the understanding of possible alterations and vulnerabilities within different communities [16,17]. Recent studies have demonstrated that mammal communities with similar environmental conditions (e.g., habitat and climate) show consistent composition in functional traits and respond similarly to drivers of change, even hosting taxonomically different species [13,18].

Myanmar has one of the largest remaining forest covers in mainland Southeast Asia, hosting different habitats that are considered to be biodiversity hotspots with many threatened species [19]. In the period of 1990–2000, more than 63% of the country was covered with its original forests [20]. Despite this, the deforestation rate has dramatically increased during the last few decades, with the third largest deforestation rate in the world in the period of 2010–2015 with 1.7% annual loss [21]. Wildlife communities in Myanmar are poorly studied and, with about 70% of human population living in rural areas [22], face several anthropogenic threats leading to high levels of forest resource exploitation, with the consequent fragmentation and degradation of habitats, as well as hunting pressure on wildlife. Moreover, it is common practice to hunt mammals and birds classified as endangered and/or rare species for personal consumption, village markets, or (worse) to feed international illegal wildlife trade [23,24]. It is therefore essential to understand the conservation status of mammal communities in these areas at a broad scale and in different habitat conditions, since very little is known about the effects of habitat degradation and hunting on species richness.

In this study, we focused on two poorly studied areas of Myanmar with two different conditions: one area in Sagaing (North Myanmar) is located inside a protected area (PA), with an efficient management regime and a well-preserved continuous evergreen forests. The other, in Rakhine (West Myanmar), is close to the border of a PA and located in a coastal area, with a strong presence of human activity in the surrounding forests [25]. Both areas support a rich biodiversity including key species for the maintenance of ecosystems like large herbivores such as the Asian elephant (*Elephas maximus*) or gaur (*Bos gaurus*), apex predators such as Indochinese tiger (*Panthera tigris*), two bear species (sun bear (*Helarctos malayanus*) and Asiatic black bear (*Ursus thibetanus*)), and two critically endangered pangolins species (*Manis javanica* and *Manis pentadactyla*) [26,27]. Some areas in Myanmar, including the two in this study, are still considered to host a potential range for many species, but the reality is that no data can confirm this fact; for wide areas, the situation is unknown. To fill this lack of knowledge, we used the camera-trapping data of medium- and large-bodied mammals collected over three consecutive years (2017–2019) in the two study areas by using the same structured monitoring scheme. Using a hierarchical modeling framework with a Bayesian formulation [28,29], we focused on the estimation (while accounting for imperfect detections [28]) of the following parameters: (1) estimated species richness for the whole community used as a proxy of species diversity and representing the most direct measurement of biodiversity [30] and (2) estimated species richness and proportion within the community of two important functional traits such as trophic niches and body mass. These two traits were largely used in these models to evaluate the general community functional composition [13,31,32]. Finally, we also focused on (3) multi-season occupancy estimates since this parameter is widely used as a proxy for population density that often is difficult, if not impossible, to measure [33].

Since we focused on two different areas with different levels of anthropogenic pressure, we expected that the fragmented and altered forest habitats hosted a lower mammal diversity and that the community would show possible alterations in the number of species, in some ecosystem functions, and/or in species abundance (i.e., lower occupancy values).

## 2. Materials and Methods

### 2.1. Study Area

**Figure 1:**
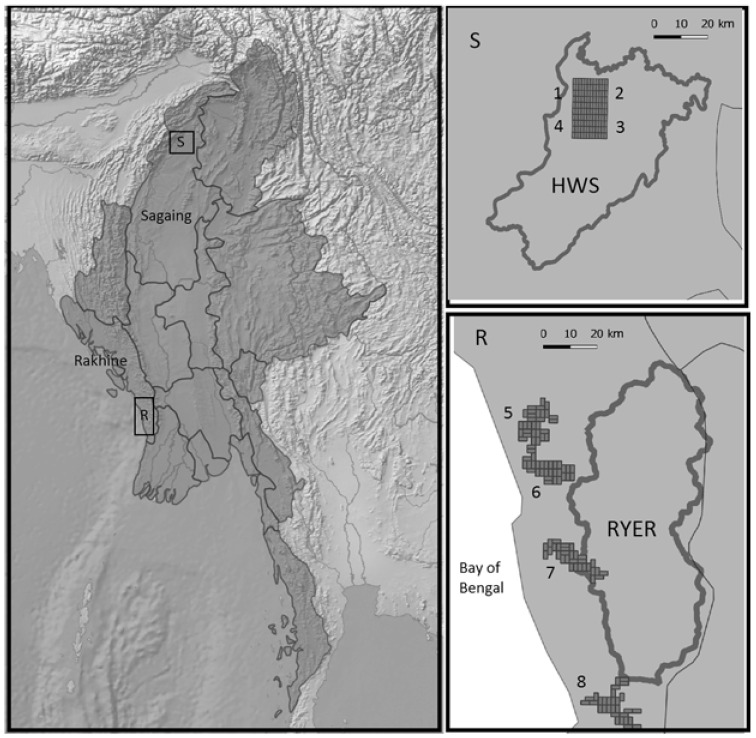
Study area locations in Myanmar (left): **S.** Detailed map of the sites in the Htamanthi Wildlife Sanctuary (HWS) (1, 2, 3, and 4), where dark grey represents the protected area borders and the grids represent the different allocations of the camera trap deployment. **R.** Detailed map of the sites in the Rakhine Yoma Elephant Range (RYER) (5, 6, 7, and 8), where dark grey represents the protected area borders, and the grids represent the different allocations of the camera trap deployments. Light grey represents states of Myanmar divided by continuous lines.

We worked in two different regions of Myanmar about 1000 km away from each other. In Sagaing State (North Myanmar), we selected a study area inside the Htamanthi Wildlife Sanctuary (HWS) in the Homalin and Hkamti townships (25°37′39″ N, 95°54′23″ E; see Figure 1). The area is mostly covered by a well-preserved evergreen forest and is part of the Northern Forest Complex connected to India [26]. The area supports a rich biodiversity, and it is subject to a low impact by human activities. Indeed, human presence is mostly located outside and around the border of the PA, with only a few records of illegal activities inside [27]. The second study area is located in Rakhine State (West Myanmar) close to the border of the Rakhine Yoma Elephant Range Wildlife Reserve (RYER) in Gwa and Thandwe townships (17°22′0″ N, 94°36′0″ E, Figure 1). Patches of evergreen forest alternate with stretches of degraded forest and bamboo breaks. The area is densely populated (88 people × km^2^ in Rakhine and 54 people × km^2^ in Sagaing; numbers gathered from the Myanmar Information Management Unit) due to its position close to the coast, where most of the settlements are concentrated and people are heavily exploited forest resources [27]. The more serious threats posed by humans are illegal logging for timber and cropland expansion, but there has also been a sizable impact from illegal hunting for bushmeat consumption and wildlife trade. The biodiversity of the two areas is known to be high and variegated [26,34] with many shared species, as well as some differences given by the geographical distance and landscape features. Both areas have been poorly studied: in particular, there have been few studies on the possible impacts of the constant and intense human presence in the RYER [25].

### 2.2. Monitoring Plan

A camera-trap monitoring plan was carried out during the dry season in Myanmar from November to April in the period of 2016–2019 (three different campaigns). For each study area, we selected four survey sites (eight in total), and each site was composed of 30 cells of 2 × 1 km with at least one camera trap per 2 km^2^ cell (120 cameras per area each year). The rectangular cell design was selected while taking environmental variable homogeneity into account, with the aim of facilitating the access in the denser forest patches. In the RYER, the difficulty in accessing forest made the sites more spaced between each other, mostly following the rivers to reach inside the forest to place cameras. In the HWS, the sites are contiguous since the area inside the PA is mostly covered by a homogeneous habitat. We randomly placed cameras within each grid cell, but we checked each camera position in the field using a GPS device (Garmin GPSMAP 64s; average positioning error of *±*10 m) to maintain a reasonable distance between them (at least 1 km). Since we had 60 cameras (Acorn Ltl-5210, Des Moines, IA, USA) for each area, we concurrently monitored two areas in the RYER and two in the HWS and then moved the cameras to the other grids. We left cameras in place for a minimum of 45 days in each site. Cameras were placed at 60 cm above the ground and set up as follows: (1) 20 s videos, with a two-minute-interval between consecutive videos; (2) a 640 × 480 pixel resolution; and (3) a passive infrared sensor (PIR) sensitivity set as “medium” with side PIR sensors active. Cameras were active 24 h per day, and they were not checked until the end of the survey period to avoid disturbance. No bait or attractant were used.

At the end of each survey, all videos collected from each camera were stored on a dedicated network attached storage server (Synology RS2416+, New Taipei City, Taiwan, 43.5 TB total storage space,) and hierarchically organized in separate folders by study area, sampling site, and camera [35]. For video content identification, we removed all videos caused by false triggers or video containing unidentifiable species and non-target species such as all birds, reptiles, small mammals (e.g., rodents), and domestic animals.

### 2.3. Data Analysis

We used two different multi-season hierarchical models to evaluate species richness and occupancies for the two communities. First, at the community level, to compare the estimated species richness of each community, we used a multi-season multi-species occupancy analysis following Rovero and Zimmermann [36]. We included all the target species (medium-large mammals) as part of the community in this analysis, and we excluded humans from the dataset. Second, at the single species level, to evaluate species abundance, we used a single species multi-season occupancy analysis [37], only for a subset of species present in both areas with at least 10 detections (lowest threshold to obtain reliable and robust models). Since the monitoring plan was carried out in both study areas during the same season and since, in addition, we did not record any relevant environmental change (e.g., fires and clearings) across years or sampling sites, we decided to pool the annual data in each area in the following analysis.

The first model (Supplementary Text S1) was used to estimate community level parameters (richness, colonization, and persistence) and derived measures of community size and structure while taking imperfect detection into account. We prepared first yearly matrices with detections *d_ij_* for each species *i* in each sampling location *j* since all the three years had the same set of species (we added rows with zeroes if a species was not detected in a given year). We used data collected by camera traps that worked in all three monitoring seasons (107 in the RYER and 108 in the HWS). We then summarized the number of detections for each species in each year with a 3D matrix *Y_i,j,t_*, where *t* represents the sampling year. The analysis also required the number of camera days by sampling unit (i.e., the sampling effort) for each year (*K_j,t_*); we used a sampling occasion of five days [32,35], which resulted in 13 occasions in the RYER and 11 in the HWS. We decided to augment the detection data of each community so that the total number of species was *M* = 100 + the number of observed species [38]. It is suggested to use this technique [39] since the dimension of the parameter vector for community size N can change at every iteration. The data augmentation involved the addition of an arbitrary number of all-zero trap frequencies considered as potentially unobserved species to the detection matrix. Then, an indicator *ώ_i_* was added when a species of the augmented dataset represented a species of the community or not. Considering *ώ_i_* as a Bernoulli-distributed random variable, i.e., *ώ_i_* ~ Bern (*Ω*), the model estimated the probability *Ω* that the species could be a member of the community of size *N*. For each year *t*, we defined an incidence matrix *Zt* with dimension *N* × *j*. Elements of the augmented matrix *Z* (of dimension *M × j*) indicated whether species *i* was present at site *j* (*z_ijt_* = 1) or not (*z_ijt_* = 0).

As described by Royle and Dorazio [40], in this model, changes in species occurrence depend on species- and site-specific persistence and colonization probabilities, and both are modeled using a first-order Markov process [41]. The initial occurrence state at *t* = 1 of species *i* at site *j* is defined as *z_ij1_* ~ Bern (*ψ_ij1_ ώ_i_*), where *ψ_ij1_* is the probability that a species *i* occurred at site *j*. The state in the other seasons *t* = 2 and *t* = 3 depend on the states at *t* − 1: *z_ij,t+1_* ~ Bern (*π_ijt_ ώ_i_*), where

π_*ijt*_ = φ_*ijt*_ z_*ijt*_ + γ_*ijt*_ (1 − z_*ijt*_)

where *φ_ijt_* is the persistence at a site (probability that a site occupied at time *t* remains occupied at *t* + 1 by species *i*) and *γ_ijt_* is the local colonization (probability that a site not occupied at time *t* becomes occupied at *t*+1 by species *i*).

Occurrence probabilities for *t* = 2 and *t* = 3 are expressed as a function of persistence and colonization

*ψ*_*ij,t*+1_ = *φ_*ijt*_* + *γ*_*ijt*_ (1 − ψ_*ijt*_).

The observation process follows a binomial distribution, *y_ijt_* ~ Bin (*K_jt_*; *p_ijt_ z_ijt_*), where *y* is the observed number of detection and *p* is the detection probabilities matrix. We assumed constant occupancy, detection probabilities, and colonization and persistence rates across sites, i.e.,:

logit (*ψ_ij1_*) = *b_i0_* with *b_i0_* ~ Normal (*μ_ψ__1_, σ _ψ__1_^2^*)

logit (*p_ijt_*) = *a_it0_* with *a_it0_* ~ Normal(*μ_p_, σ_p_^2^*)

logit (*γ_ijt_*) = *c_it0_* with *c_it0_* ~ Normal(*μ*_γ_*, σ*_γ_*^2^*)

logit (*φ_ijt_*) = *d_it0_* with *d_it0_* ~ Normal(*μ_φ_*, *σ*_φ_*^2^*)

We fit the model with a Bayesian formulation and Markov chain Monte Carlo (MCMC) using the JAGS software [42] ran through R [43] with the R packages *rjags* and *dclone*. We used three chains, and we set the following initial values (see Supplementary Text S1): run 50,000 times after 5000 burn-in iterations and thinning every 10 draws. We used uninformative priors and verified model convergence with the visual inspection of the chains (R package *mcmcplots*) and with the Gelman–Rubin diagnostic [44].

We also evaluated community species richness and community composition grouping species by trophic niches according to the database provided by Wilman et al. [45]. The selected trophic niches were: “herbivore,” “carnivore,” “omnivore,” and “insectivore.” We also divided mammals in different body mass groups to add another functional trait in the comparison of the two community compositions: we grouped mammals into “medium”-sized (1 kg < body mass < 10 kg), “medium-large”-sized (10 kg < body mass < 100 kg), and “large”-sized (>100 kg).

For the single species multi-season occupancy analysis (Supplementary Text S2), we constructed a list with all the detection (1) non-detection (0) observations for all the selected species for each year. We then separately ran the multi-season model for each species, for all species with at least 10 detections in both areas. Each single species list was created so that all years contained the same number of camera traps location (rows) and sampling occasion (columns), with the sampling session defined as a five day interval (reasonable average for medium-large mammals [46]) and the year with less sampling occasions determining how many observations to be extracted from the other two years. We arranged single-species data as 3D arrays, *Y_i,j,t_,* where *i* represents site, *j* is the sampling occasion, and *t* is the year. True occurrence was modeled as a Bernoulli-distributed random variable *z_i,t_* ~ Bern (*ψ_i,t_*) with probability *ψ_i,t_* (*z* = 1) when species were present at site *I* and year *t*.

We modeled occupancy and detection probabilities across sites as logit (*ψ_ij_*) = *b_i0_* and logit (*p_ij_*) = *a_i0_* with *b_i0_* ~ Normal(*β_0_, σ_b0_^2^*) and *a_i0_* ~ Normal (*α_0_, σ_a0_^2^*). We included parameters of persistence and colonization (see richness section above) in the model, and we fit the model with a Bayesian formulation with the same setting and convergence validation used for the species richness analysis (see Supplementary Text S2).

## 3. Results

Overall, the monitoring effort across three years involved 108 cameras in the HWS (324 total) and 107 in the RYER (321 total), with cameras operating for an average of 55 and 66 days, respectively. The total number of used videos (i.e., excluding non-target species and false trigger videos) collected in three years for both areas was 9529, 6324 of which were of medium-large mammals and 3205 were of humans. The number of videos for target mammals and humans varied between the two areas. In the RYER, we obtained 639, 954, and 885 mammal videos, respectively, during the three years, and 869, 952, and 1288 vides of human activities; meanwhile in the HWS, 1309, 1218, and 1319 videos were of mammals and 25, 37, and 34 were of humans.

### 3.1. Species Richness

In the HWS, cameras detected 32 different species (year 1: 26; year 2: 28; and year 3: 24), and we detected 25 different species in the RYER (year 1: 20; year 2: 21; and year 3: 20). The two communities presented a large part of shared species (22): in particular, almost all the RYER species were present in the HWS, but the contrary did occur (see Table S1).

**Figure 2:**
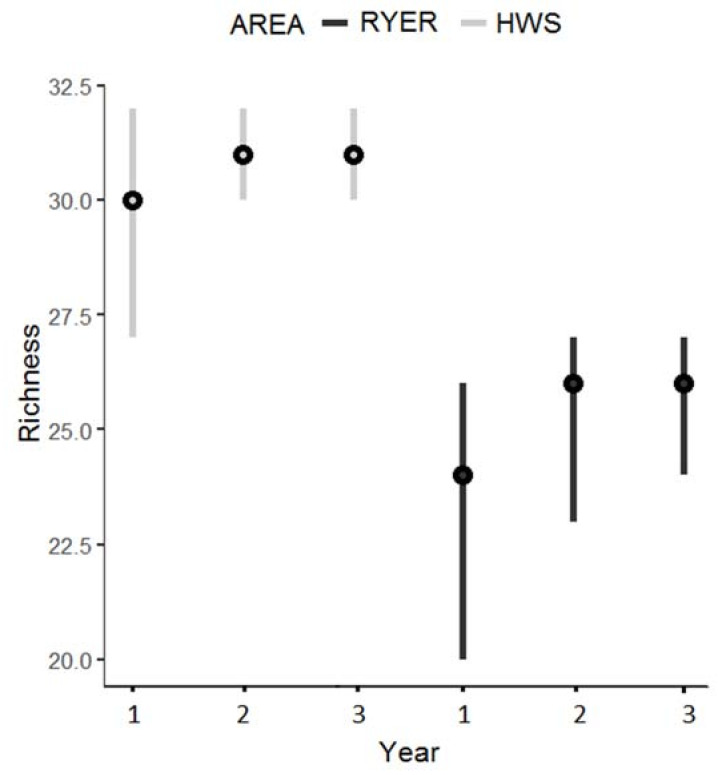
Estimated species richness during the 3 years of monitoring in both areas (Htamanthi Wildlife Sanctuary (HWS): light grey; Rakhine Yoma Elephant Range (RYER): black) with median (black dots) and 95% Bayesian credible intervals.

Estimated species richness differed between areas with all the 95% Bayesian credible intervals (BCIs) significantly different. In the HWS in the first year, the estimated median species richness was 30 (mean 29.93; 95% BCI 27–32), 31 for the second year (mean 30.91; 95% BCI 30–32), and 31 for the third year (mean 30.99; 95% BCI 30–32). In the RYER, the species richness estimates for the three years were 24, 26, and 26, respectively (means: first year 23.27 and 95% BCI 20–26; second year: 25.27 and 95% BCI 23–27; and third year: 25.71 and 95% BCI 24–27) (Figure 2).

The total estimated richness, considering all years, was 31 (mean 31.17; 95% BCI 31–32) for the HWS and 26 (mean 26.22; 95% BCI 26–28) for the RYER. Table 1 reports the rate of colonization and persistence between years: both areas showed significantly higher values of persistence compared to colonization. The parameter ώ (that is the probability for a species in the augmented dataset to be part of the community) was similar between areas with 0.24 (0.17–0.32) for the HWS and 0.21 (0.15–0.29) for the RYER.

**Figure 3:**
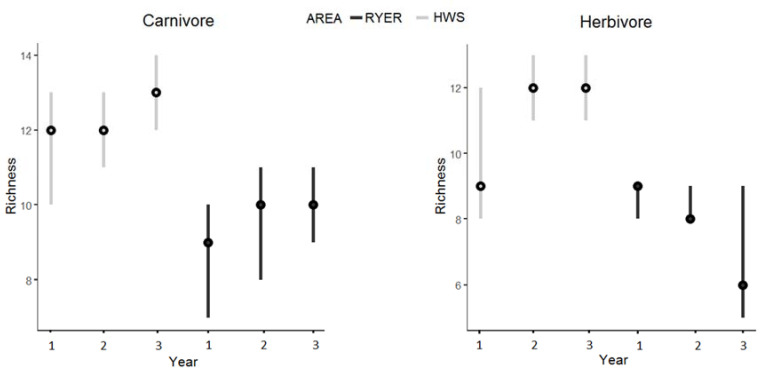
Estimated species richness during the 3 years of monitoring in both areas (Htamanthi Wildlife Sanctuary (HWS): light grey; Rakhine Yoma Elephant Range (RYER): black) with median (black dots) and 95% Bayesian credible intervals for the two significantly different groups of carnivore and herbivore.

Regarding community structure, (trophic niches; Table S1), we found similar patterns. The HWS showed a greater richness for carnivores and herbivores (carnivores: 13 (13–15) for the HWS and 10 (10–11) for the RYER; herbivores: 12 (12–13) in the HWS and 9 (9–10) in the RYER (Figure 3)), with almost all the 95% BCIs significantly different between areas and among years (Table 2).

**Figure 4:**
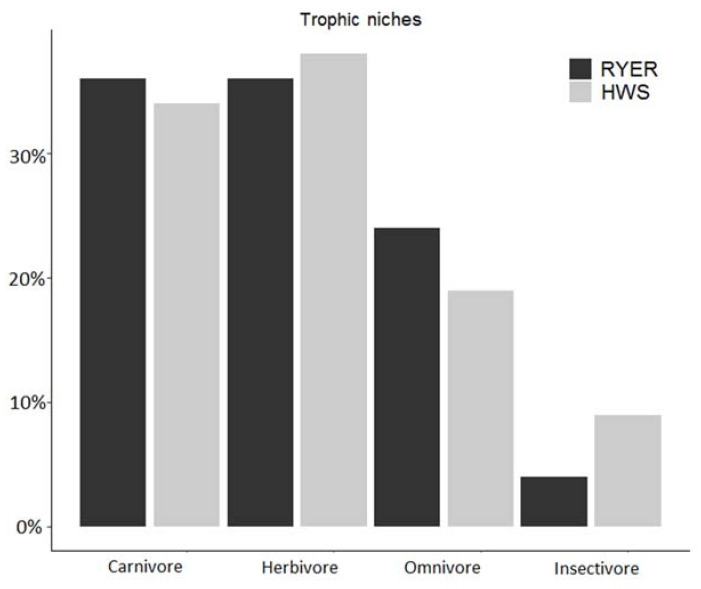
Proportion in % of each trophic niche compared to the total number of observed species in each community (Rakhine Yoma Elephant Range (RYER): black; Htamanthi Wildlife Sanctuary (HWS): grey).

The only non-significant difference between the two study areas was for the omnivore group. No result was obtained for the insectivore niche, as we found few species with low detection numbers and the model did not converge. We recorded only one “true” insectivore (in the ecological sense of the term) in the RYER (Sunda pangolin) and three insectivores in the HWS (both pangolin species and the hog badger, *Arctonyx collaris*). The proportion of each trophic niche in both communities was similar for carnivores (36% in the RYER and 34% in the HWS) and herbivores (36% in the RYER and 38% in the HWS), whereas small differences were found for omnivores (24% in the RYER vs. 19% in the HWS) and insectivores (4% in the RYER vs. 9% in the HWS) (Figure 4).

**Figure 5:**
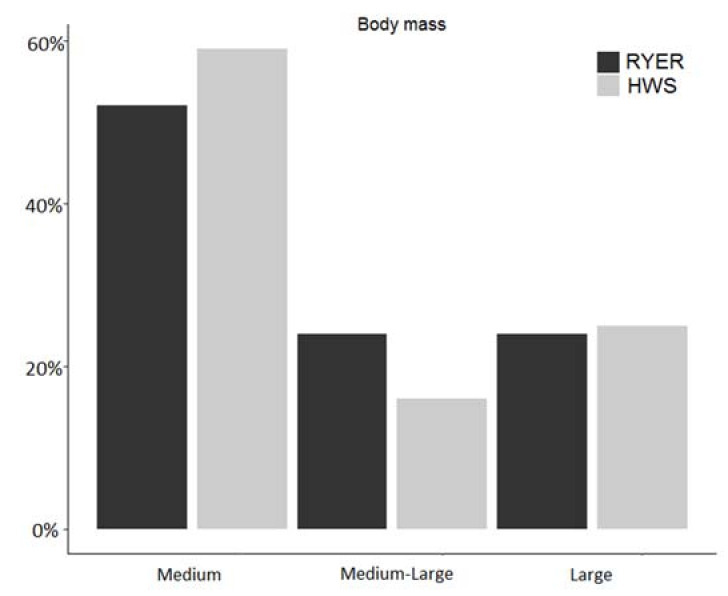
Proportion in % of each body mass groups compared to the total number of observed species in each community (Rakhine Yoma Elephant Range (RYER): black; Htamanthi Wildlife Sanctuary (HWS): grey).

When body mass was taken into account as a further functional trait, we found similar patterns for large-sized mammals (20% in the RYER and 22% in the HWS), slight differences for medium-sized mammals (52% in the RYER vs. 59% in the HWS), and a remarkable difference for the medium-large mammals group (28% in the RYER vs. 19% in the HWS) (Figure 5). Table 3 shows both functional traits together, along with the number of species for each trophic niche and body mass categorization.

### 3.2. Occupancy

**Figure 6:**
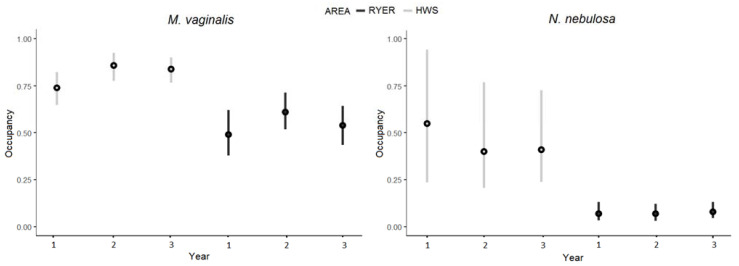
Occupancies values (y axis) for *Muntiacus vaginalis* and *Neofelis nebulosa* with median (black dots) and 95% Bayesian credible intervals for the three years of monitoring (x axis) in the two areas (grey: Htamanthi Wildlife Sanctuary (HWS); black: Rakhine Yoma Elephant Range (RYER)).

As can be seen from Table S2, the estimated occurrence probabilities were generally higher in the HWS than in the RYER, with significant differences between areas for some species. In particular, the Northern red muntjac (*Muntiacus vaginalis*) and the clouded leopard (*Neofelis nebulosa*) had occurrence differences between areas for all three years, with higher values in the HWS (Figure 6). The Northern red muntjac also had significantly higher detectability values in the HWS, while we found an opposite result for humans (higher detectability in the RYER). Finally, we found significant differences in the persistence and colonization (Table S3) parameters for the clouded leopard, and the Northern red muntjak showed higher persistence values for both areas. For all other species, we estimated highly variable occupancy values, as well as unstable persistence–colonization rates across the years. In this analysis, we also estimated occupancy values for humans and found a significant difference between areas, especially in the first year, with generally higher values for the RYER (Table S2).

## 4. Discussion

Analyzing camera trap data at the community level across study areas is useful in understanding the status of communities between countries or different regions [18]. In this study, we analyzed the status of two mammal communities in two geographically distinct areas in Myanmar that are similar in climate, elevation, and habitat types but have sharp differences in their levels of habitat degradation and human disturbance. We used camera trap data to evaluate both biodiversity levels (estimated species richness) and some ecosystem functions such as trophic niches and body mass in two different contexts: a well-preserved and mostly untouched evergreen forest and a degraded and fragmented forest with a high level of human pressure [25]. It is important to increase our knowledge of the status of communities and species because no previous studies, with a structured monitoring scheme, have been done in these study areas. There is a need to understand the priority area for conservation and key species on which to concentrate efforts. Documenting the status of two mammal communities in a poorly studied country like Myanmar, we found patterns that were comparable with other studies in tropical forest communities in terms of the number of species [13,32,47]. Surprisingly, we found two similar communities in terms of shared species (22), despite the distance between the two study areas, with only a few differences depending on different geographical ranges. As we can see from the detection list, two of the species detected only in the RYER (golden jackal (*Canis aureus*) and Phayre’s leaf monkey (*Trachypithecus phayrei*)) were not previously recorded in North Myanmar. For the species detected exclusively in the HWS, instead, we found some species with a specific distribution in the north of the country (Shortridge’s langur (*Trachypithecus shortridgei*) Rhesus macaque (*Macaca mulatta*), and Stump-tailed macaque (*Macaca arctoides*)) and some others that were historically known as present for the RYER but were not recorded during our monitoring, like the endangered Indochinese tiger [48] and medium body-sized mammals like the Chinese pangolin, the yellow throated marten (*Martes flavigula*), and the back-striped weasel (*Mustela strigidorsa*) [49,50,51], as well as two badger species: the Burmese ferret-badger (*Melogale personata*) and the hog badger [52]. For the Chinese serow (*Capricornis milneerdwadsii)*, there was uncertainty due to the lack of studies on the species distributions in Myanmar.

Using estimated species richness as a proxy for biodiversity [30], we found that the disturbed area in the RYER hosts a lower diversity in species, both for the whole community and for the functional trophic niches of carnivores and herbivores. Many studies have confirmed that the alteration of forest habitat has a depleting effect on mammal communities [3,8,9,10], especially on carnivores and herbivores species. Carnivores have been documented as suffering a huge decline in their geographical range [53], but herbivores are also facing high extinction rates at the global scale [31]. We did not find significant differences for omnivore groups, showing a similar number of species in both areas. Roemer et al. [54] pointed out that medium-sized omnivores are the more adaptable and opportunistic species group, living in a wide range of habitats, even with high level of anthropogenic disturbances. We were not successful in estimating species richness for insectivores, as we had an insufficient number of detections in the RYER (only one species: Sunda pangolin) that did not allow for the occupancy models to converge. We are confident that even this could be considered to be a result. In the HWS, we found three insectivore species (the two pangolins species and the hog badger), which was a greater species richness compared to the first area for a group known to be particularly sensitive to habitat disturbance worldwide [32,55] while also considering that the distribution of these species should also have included the RYER. We are aware that a direct comparison between areas could be complicated because it can be reductive to reduce differences to anthropogenic disturbance, since many factors can come into play like the different landscape features given by the large distance between areas. However, it is a fact that the human disturbance found in the RYER, both in terms of human presence in the forests and the level of degradation, had a large and significant differences between areas having a possible influence on the evaluated parameters. We retained these very important data to increase our knowledge of the patterns of occurrence of different wildlife species on two understudied areas in Myanmar.

If we look at the proportion of each group in the community, the structure and functional composition seemed similar. The disturbed area hosted a lower species number but did not show any alteration in the functional composition of the community. All the specific roles in the RYER were present, with one exception. The first thing that stands out, is the lack of detection of large carnivores in this area. During our three years of monitoring, we found large-sized (>100 kg) carnivores (tiger) only in the HWS (not in the RYER), even if potential prey, such as large-bodied herbivores such as the sambar deer (*Rusa unicolor)*, gaur, the red serow *(Capricornis rubidus),* or even Asian elephants were present. All these species are too big for medium-large carnivores such as the clouded leopard or the Asiatic golden cat (*Catopuma temminckii),* leaving only the dhole (*Cuon alpinus*) able to exploit large herbivores, except for elephants, as a species hunting in packs [56,57]. Historical data supported the presence of tigers in the RYER [48], and leopards are known to be present inside the Yoma Elephant Range [58], but we did not detect either species during our three years of monitoring. In the HWS, instead, we documented the presence of tigers, and we also know that leopards are commonly present [26,59]. This result suggests a possible effect of human disturbance on large carnivores in the RYER, as already documented in many other areas of the world [60,61,62,63]. Certainly, this fact highlights the importance of focusing conservation efforts on this threatened group because human–large carnivore conflict is one of the main reason of the decline of this group worldwide [64], especially for tigers [65]. In the area of the HWS, there is a conservation program for tigers supported by the Wildlife Conservation Society; it would be interesting to develop one in the region of the RYER to better understand the status of these key species for the ecosystem.

Trophic niches can be used to understand the various roles in ecosystem functions [31]. Carnivores and herbivores were found to occupy the largest proportion of the communities in both areas. This pattern frequently occurs and has been reported in other studies, though with the largest share of the community occupied by herbivores [32]. We were surprised in finding such a high proportion of carnivores, especially for meso-carnivores. A tentative explanation is that a large part of potential prey was represented by small mammals (<1 kg body mass), such as Sciuridae or Muridae, that were not considered in this analysis, being mostly below the detectability level of a camera trap system focused on medium-large mammals. In addition, it could be possible that the absence of large carnivore released the underlying groups, as in the medium carnivore group. This would not detract from the obtained results, since the stronger human pressure in Myanmar mostly occurs for herbivore species such as the Northern red muntjak, gaur, or sambar deer for local bushmeat consumption in villages [23,24]. It is only a speculation, but it would be interesting to investigate whether humans can “play” the role of apex predators with high rates of hunting in disturbed areas, as we found in our area.

The body mass parameter is often used as a proxy to check for responses to changes in the community [15], and we found, as expected, that the largest part of both communities were occupied by medium-sized mammals, followed by medium-large and large mammals. If the proportion of the community occupied by large mammals was surprisingly the same in the RYER and the HWS (apart from the difference in large carnivores—there was none for herbivores), the proportion of medium-large mammals was quite different, with higher values in the RYER than in the HWS, even if the number of different species was similar. This was likely due to the absence of large carnivores, as explained above, or by the higher proportion of medium-sized mammals in the HWS community with respect to the medium-large mammals. In addition, it would be interesting to evaluate the small mammal community present in the RYER that was not the target of this work but could support high numbers of medium-sized mammals like meso-carnivores, as explained above. Though it is unquestionable that we found a lower diversity in the RYER, the functional composition of the two communities was similar, not showing any evident alteration in the considered traits (body mass and diet). As already found in other similar works, it seems that tropical forests communities show a consistent functional trait composition around the world, although with different taxa [13,18].

Occupancy estimates at the species level, intended as a proxy for density [33], showed generally lower values for the RYER for all the three years, with significantly lower values for the Northern red muntjak and the clouded leopard. Many models present a great variability in BCIs that could probably be explained by the fact that many species had too few detections to converge in the model, and occupancy results were therefore considered as unreliable for some species. For the two mentioned species, the first was widely occurring (the most detected after humans) but also a target species for hunting activities and bushmeat consumption [24]. Meanwhile, the clouded leopard is a vulnerable species that is threatened by habitat degradation and hunting for the illegal wildlife trade [24]. In addition, these two species constitute a predator–prey system, with the Northern red muntjak one of the main prey for clouded leopards [66,67,68]. We did not record differences between areas for large-bodied mammals (except for apex predators), even if we expected it, but all the species in this group had the lowest occupancy values in both areas. An explanation could be that these species, due to their large size, are more affected by habitat degradation and coexistence with humans over time [69,70,71].

The colonization and persistence rates for the Northern red muntjak and clouded leopard were significantly different. The rates were higher for the latter, suggesting a stability of the species in the area across years. All other species occurrence values were widely variable across the years to allow for the detection of a trend for the populations, even though three years was quite a small interval to base assessments of temporal change. It appears from our study that a single season monitoring scheme could have misleading results. A multiple-season approach gives more robust results in community studies, which are useful when addressing multiple years of monitoring general population trends.

A separate consideration concerns humans, whose presence was recorded by a considerably higher number of detections in the RYER (3109) compared to the HWS (96), with significantly higher occurrence and detectability values. This fact confirmed that the RYER is more disturbed, dealing with not only “chronic” disturbance effects like habitat degradation and fragmentation but also “acute” disturbance conditions due to the high rate of human presence in the forests that can affect species in many ways, as documented for this area with a significant effect on some species activity patterns [25]. As we said previously, we are aware that the two areas were distant from each other, meaning that some community patterns could be related to differences in geographical and landscape features and not only to human disturbances. Dealing with community analysis, and consequently a wide range of species occupying various trophic levels, we have to consider that differences in the faunal composition could be attributable to many factors from environmental to anthropogenic. It will be interesting to investigate this specific fact in the future and to specifically focus on the RYER area in order to consider whether the different human activities and/or some environmental covariates affect species’ occupancy and detectability and on which species the pressure is stronger. It is evident that there could be a potential effect of human disturbance on some species, but considering the whole community, a wide spectrum of single species with different characteristics and possible reactions should be taken into account. In this work, the focus was to increase knowledge at a community level, but we are now also investigating these possible effects because they require different analyses and species-specific levels of detail and depth.

## 5. Conclusions

In conclusion, we believe this is an important documentation of the status of two different communities in a poorly studied area as Myanmar. We now have a picture of the situation of the two communities in terms of the number of species and faunal composition to better address governments and stakeholders for specific conservation actions on these areas, as already done for the sun bear species with the development of a ten year conservation plan for the two study areas [27]. We can affirm that our hypothesis of a lower mammal diversity in a disturbed area with high levels of fragmentation and exploitation by human activities was confirmed, but we did not find a significant alteration of the functional roles in the community, except for apex predators. This fact should be better investigated with specific monitoring plans to understand whether the absence of large carnivores was due to our lack of detection or if the species are extinct in the study area. Furthermore, our existing dataset allows for the continued analysis of direct and indirect human activities while trying to understand their impact on single species, especially for target species for hunting. It is indeed documented that illegal activities, such as hunting or logging, may affect mammal diversity and abundance when occurring for a long time, with a consequent change in habitat structure that could possibly lead to local extinctions [72,73,74].

## Figures and Tables

**Table 1:** Rate of colonization and persistence of the two communities (Rakhine Yoma Elephant Range (RYER) and Htamanthi Wildlife Sanctuary (HWS)) between the first and second years (1) and between the second and third years (2); ώ represents the probability that a species of the augmented dataset is part of the community. For all the parameters, median values with 95% Bayesian credible intervals (BCIs) are reported. Asterisks denote that persistence and colonization values were significantly different (no overlap between BCIs).

Areas	Colonization (1)	Persistence (1)	Colonization (2)	Persistence (2)	ώ
RYER	0.09 (0.03–0.24)	0.55 (0.36–0.72) *	0.08 (0.02–0.21)	0.54 (0.35–0.72) *	0.21 (0.15–0.29)
HWS	0.13 (0.04–0.30)	0.62 (0.42–0.80) *	0.15 (0.04–0.35)	0.69 (0.49–0.85) *	0.24 (0.17–0.32)

**Table 2:** Estimated species richness (median and 95% Bayesian credible intervals) for the three considered trophic niches groups (herbivore, carnivore, and omnivore) in the two areas (Htamanthi Wildlife Sanctuary (HWS) and Rakhine Yoma Elephant Range (RYER)) during the three years (y1, y2, and y3). “tot” indicates the total estimated species richness.

Trophic Niches	HWS y1	HWS y2	HWS y3	RYER y1	RYER y2	RYER y3	HWS tot	RYER tot
Herbivore	9 (8–12)	12 (11–13)	12 (11–13)	9 (8–9)	8 (8–9)	6 (5–9)	12 (12–13)	9 (9–10)
Carnivore	12 (10–13)	12 (11–14)	13 (12–14)	9 (7–10)	10 (8–11)	10 (9–11)	13 (13–15)	10 (10–11)
Omnivore	5 (5–8)	6 (4–8)	6 (4–8)	6 (5–25)	6 (6–25)	6 (6–25)	6 (6–9)	6 (6–25)

**Table 3:** Number of observed species for each trophic niche group (carnivore, herbivore, omnivore, and insectivore) in each body mass group (M: medium; M-L: medium-large; L: large) in both areas (left in white: Rakhine Yoma Elephant Range (RYER); right in grey: Htamanthi Wildlife Sanctuary (HWS)).

Trophic Niches/ Body Mass	M	M-L	L	M	M-L	L
Carnivore	*Herpestes urva* *Pardofelis marmorata* *Prionailurus bengalensis* *Prionodon pardicolor* *Viverra zibetha*	*Canis aureus* *Catopuma temminckii* *Cuon alpinus* *Neofelis nebulosa*	/	*Herpestes urva* *Martes flavigula* *Mustela strigidorsa* *Pardofelis marmorata* *Prionailurus bengalensis* *Prionodon pardicolor* *Viverra zibetha*	*Catopuma temminckii* *Cuon alpinus* *Neofelis nebulosa*	*Panthera tigris*
Herbivore	*Atherurus macrourus* *Hystrix brachyura* *Macaca leonina* *Trachypithecus phayrei*	*Muntiacus vaginalis*	*Bos gaurus* *Capricornis rubidus* *Elephas maximus* *Rusa unicolor*	*Atherurus macrourus* *Hystrix brachyura* *Macaca leonina* *Macaca arctoides* *Macaca mulatta* *Trachypithecus shortridgei*	*Muntiacus vaginalis*	*Bos gaurus* *Capricornis rubidus* *Capricornis milneedwardsii* *Elephas maximus* *Rusa unicolor*
Omnivore	*Arctictis binturong* *Paradoxurus hermaphroditus* *Viverricula indica*	*Sus scrofa* *Helarctos malayanus*	*Ursus thibetanus*	*Arctictis binturong* *Melogale personata* *Paradoxurus hermaphroditus*	*Sus scrofa* *Helarctos malayanus*	*Ursus thibetanus*
Insectivore	*Manis javanica*	/	/	*Arctonyx collaris* *Manis pentadactyla* *Manis javanica*	/	/

## Data Availability

The datasets analyzed during the current study are available from the corresponding author on reasonable request.

## Supplementary Materials

The following are available online at https://www.mdpi.com/2076-2615/11/3/880/s1, Supplementary Text S1—R and JAGS code for the multi-seasons, multi-species community model fitted to detection/non-detection data; Supplementary Text S2—R and JAGS code for the multi-seasons, single-species occupancy model fitted to detection/non-detection data; Table S1—Checklist of medium-large mammals detected by camera traps; Table S2—Occupancy and detectability values in the three years of monitoring for the selected species in the two areas; Table S3—Colonization and persistence values between years for the selected species in the two areas.
