# Supplementary material for: Camera Trapping to Assess Status and Composition of Mammal Communities in a Biodiversity Hotspot in Myanmar
Source: Animals (Basel). 2021 Mar 19;11(3):880. doi: 10.3390/ani11030880 (PMC8003726; doi:10.3390/ani11030880)
Supplement: Supplementary file 1 [file animals-11-00880-s001.pdf]

Supplementary Text S1. R and JAGS code for the multi-seasons, multi-species community model fitted to detection/non-detection data.

```
# model
modelFilename = "richness.txt"

cat("
    model {
    # priors
    omega ~ dunif(0,1)
    psiMean ~ dunif(0,1)
    for (t in 1:T) {
    pMean[t] ~ dunif(0,1)
    }
    for (t in 1:(T-1)) {
    phiMean[t] ~ dunif(0,1)
    gamMean[t] ~ dunif(0,1)
    }
    lpsiMean <- log(psiMean) - log(1-psiMean)
    for (t in 1:T) {
    lpMean[t] <- log(pMean[t]) - log(1-pMean[t])
    }
    for (t in 1:(T-1)) {
    lphiMean[t] <- log(phiMean[t]) - log(1-phiMean[t])
    lgamMean[t] <- log(gamMean[t]) - log(1-gamMean[t])
    }
    lpsiSD ~ dunif(0,10)
    lpsiPrec <- pow(lpsiSD,-2)
    lpSD ~ dunif(0,10)
    lphiSD ~ dunif(0,10)
    lgamSD ~ dunif(0,10)
    for (t in 1:T) {
    lpPrec[t] <- pow(lpSD,-2)
    }
    for (t in 1:(T-1)) {
    lphiPrec[t] <- pow(lphiSD,-2)
    lgamPrec[t] <- pow(lgamSD,-2)
    }

    # likelihood
    for (i in 1:M) {
    # initial occupancy state at t=1
    w[i] ~ dbern(omega)
    b0[i] ~ dnorm(lpsiMean, lpsiPrec)T(-12,12)
```

```
lp[i,1] ~ dnorm(lpMean[1], lpPrec[1])T(-12,12)
p[i,1] <- 1/(1+exp(-lp[i,1]))
for (j in 1:n.site) {
lpsi[i,j,1] <- b0[i]
psi[i,j,1] <- 1/(1 + exp(-lpsi[i,j,1]))
mu.z[i,j,1] <- w[i] * psi[i,j,1]
Z[i,j,1] ~ dbern(mu.z[i,j,1])
mu.y[i,j,1] <- p[i,1]*Z[i,j,1]
Y[i,j,1] ~ dbin(mu.y[i,j,1], K_tot[j,1])
}
# model of changes in occupancy state for t=2, ..., T
for (t in 1:(T-1)) {
lp[i,t+1] ~ dnorm(lpMean[t+1], lpPrec[t+1])T(-12,12)
p[i,t+1] <- 1/(1+exp(-lp[i,t+1]))
c0[i,t] ~ dnorm(lgamMean[t], lgamPrec[t])T(-12,12)
d0[i,t] ~ dnorm(lphiMean[t], lphiPrec[t])T(-12,12)
for (j in 1:n.site) {
lgam[i,j,t] <- c0[i,t]
gam[i,j,t] <- 1/(1+exp(-lgam[i,j,t]))
lphi[i,j,t] <- d0[i,t]
phi[i,j,t] <- 1/(1+exp(-lphi[i,j,t]))
psi[i,j,t+1] <- phi[i,j,t]*psi[i,j,t] + gam[i,j,t]*(1-psi[i,j,t])
mu.z[i,j,t+1] <- w[i] * (phi[i,j,t]*Z[i,j,t] + gam[i,j,t]*(1-Z[i,j,t]))
Z[i,j,t+1] ~ dbern(mu.z[i,j,t+1])
mu.y[i,j,t+1] <- p[i,t+1]*Z[i,j,t+1]
Y[i,j,t+1] ~ dbin(mu.y[i,j,t+1], K_tot[j,t+1])
}
}
}

# Derive total species richness for the metacommunity
N_tot <- sum(w[])

# Derive yearly species richness
for (i in 1:M) {
for (t in 1:T) {
tmp[i,t] <- sum(Z[i,,t])
tmp2[i,t] <- ifelse(tmp[i,t]==0,0,1)
}
}
for (t in 1:T) {
N[t] <- sum(tmp2[,t])
}
  }
```

```
", fill=TRUE, file=modelFilename)


# number of traps
nsites <- dim(Yaug_tot)[2]

# number of primary periods (years)
T <- dim(Yaug_tot)[3]

# latent states
w <- c(rep(1,dim(y2016)[1]),rep(NA,nzeros))

# Parameters monitored
parameters <- c("omega","psiMean","pMean","phiMean","gamMean",
                "lpsiSD","lpSD","lphiSD","lgamSD",
                "N","N_tot")

# data
bugs.data <- list(M=dim(Yaug_tot)[1],n.site=nsites,K_tot=K_tot,Y=Yaug_tot,T=T)


# Initial values
inits <- function() { list(omega=runif(1),Z=(Yaug_tot>0)*1,w=w,
                          psiMean=runif(1),pMean=runif(T),phiMean=runif(T-1),gamMean=runif(T-1),
                          lpsiSD=runif(1,0,4),lpSD=runif(1,0,4),lphiSD=runif(1,0,4),lgamSD=runif(1,0,4))}

#mcmc settings
n.adapt <- 5000          #pre-burnin
n.update <- 10000 #burnin
n.iter <- 50000          #iterations post-burnin
thin <- 10
chains<-3

# run the model and record the run time
cl <- makeCluster(chains, type = "SOCK")
start.time = Sys.time()
out <- jags.parfit(cl, data = bugs.data,
                   params = parameters,
                   model = "richness.txt",
                   inits = inits,
                   n.adapt = n.adapt,
                   n.update = n.update,
                   n.iter = n.iter,
                   thin = thin, n.chains = chains)
```

```
end.time = Sys.time()
elapsed.time = difftime(end.time, start.time, units='mins')
cat(paste(paste('Posterior computed in ', elapsed.time, sep=''), ' minutes\n', sep=''))
stopCluster(cl)

###end of script
```

Supplementary Text S2. R and JAGS code for the multi-seasons, single-species occupancy model fitted to detection/non-detection data.

```
# the model
modJ.bug<- function () {

  # Specify priors
  psi1 ~ dunif(0, 1)
  for (k in 1:(nyear-1)){
    phi[k] ~ dunif(0, 1)
    gamma[k] ~ dunif(0, 1)
    p[k] ~ dunif(0, 1)
  }
  p[nyear] ~ dunif(0, 1)

  # Ecological submodel: Define state conditional on parameters
  for (i in 1:nsite){
    z[i,1] ~ dbern(psi1)
    for (k in 2:nyear){
      muZ[i,k]<- z[i,k-1]*phi[k-1] + (1-z[i,k-1])*gamma[k-1]
      z[i,k] ~ dbern(muZ[i,k])
    } #k
  } #i

  # Observation model
  for (i in 1:nsite){
    for (j in 1:nrep){
      for (k in 1:nyear){
        muy[i,j,k] <- z[i,k]*p[k]
        y[i,j,k] ~ dbern(muy[i,j,k])
      } #k
    } #j
  } #i

  # Derived parameters: Sample and population occupancy, growth rate and turnover
  psi[1] <- psi1
  n.occ[1]<-sum(z[1:nsite,1])
```

```
    for (k in 2:nyear){
        psi[k] <- psi[k-1]*phi[k-1] + (1-psi[k-1])*gamma[k-1]
        n.occ[k] <- sum(z[1:nsite,k])
        growthr[k] <- psi[k]/psi[k-1]
        turnover[k-1] <- (1 - psi[k-1]) * gamma[k-1]/psi[k]
    }
}

# Initial values
zst <- apply(J, c(1, 3), sum, na.rm=T) # Observed occurrence as inits for z
zsti<-ifelse(zst>0,1,0)
jags.inits <- function(){ list(z = zsti)}
jags.params <- c("psi", "phi", "gamma", "p", "n.occ", "turnover")

# call jags
jagsfit<-jags(data=list(y = J, nsite = dim(J)[1], nrep = dim(J)[2], nyear = dim(J)[3]), inits=jags.inits, jags.params,
                        n.burnin = 5000,n.iter=50000,n.thin = 10, n.chains=3, model.file=modJ.bug)

print(jagsfit, dig=2)
```

Table S1. Checklist of medium-large mammals detected by camera traps from 2016–2019 at Rakhine Yoma Elephant Range (RYER) and Htamanthi Wildlife Sanctuary (HWS) in Myanmar. IUCN column shows the threat category of each species (LC = least concern; NT = near threatened; VU = vulnerable; EN = endangered; CR = critically endangered). Events column indicate the total number of detection and, where necessary, also divided by areas (left RYER and right HWS). Diet and Body mass columns shows how the species were classified into functional traits (M = medium; M-L = medium-large; L = large). Area column show where the species it is supposed to be present (R = Rakhine; S = Sagaing); in red where the species was not detected although in literature it is marked as its geographic range.

Latin Name	Common Name	IUCN	Events	Diet	Body Mass	Area
Arctictis binturong	Binturong	VU	9 (8/1)	Omnivore	M	R – S
Arctonyx collaris	Hog badger	VU	14	Insectivore	M	R – S
Atherurus macrourus	Asiatic brush-tailed porcupine	LC	1047 (2/1045)	Herbivore	M	R – S
Bos gaurus	Gaur	VU	51 (37/14)	Herbivore	L	R – S
Canis aureus	Golden jackal	LC	2	Carnivore	M-L	R
Capricornis milneedwardsii	Chinese serow	NT	3	Herbivore	L	S
Capricornis rubidus	Red serow	NT	15 (14/1)	Herbivore	L	R – S
Catopuma temminckii	Asiatic golden cat	NT	101 (66/35)	Carnivore	M-L	R – S
Cuon alpinus	Dhole	EN	31 (21/10)	Carnivore	M-L	R – S
Elephas maximus	Asian elephant	EN	243 (158/85)	Herbivore	L	R – S
Helarctos malayanus	Sun bear	VU	243 (198/45)	Omnivore	M-L	R – S
Herpestes urva	Crab-eating mongoose	LC	20 (11/9)	Carnivore	M	R – S
Hystrix brachyura	Malayan porcupine	LC	623 (402/221)	Herbivore	M	R – S
Macaca arctoides	Stump-tailed macaque	VU	229	Herbivore	M	S
Macaca leonina	Northern pig-tailed macaque	VU	210 (153/57)	Herbivore	M	R – S
Macaca mulatta	Rhesus macaque	LC	16	Herbivore	M	S
Manis javanica	Sunda pangolin	CR	19 (4/15)	Insectivore	M	R – S
Manis pentadactyla	Chinese pangolin	CR	5	Insectivore	M	R – S
Martes flavigula	Yellow throated marten	LC	84	Carnivore	M	R – S
Melogale personata	Burmese ferret-badger	LC	13	Omnivore	M	R – S
Muntiacus vaginalis	Northern red muntjak	LC	1662 (638/1024)	Herbivore	M-L	R – S
Mustela strigidorsa	Back-striped weasel	LC	4	Carnivore	M	R – S

Neofelis nebulosa	Clouded leopard	VU	138 (38/100)	Carnivore	M-L	R – S
Panthera tigris	Tiger	EN	22	Carnivore	L	R – S
Paradoxurus hermaphroditus	Asian palm civet	LC	339 (130–209)	Omnivore	M	R – S
Pardofelis marmorata	Marbled cat	NT	55 (15–40)	Carnivore	M	R – S
Prionailurus bengalensis	Leopard cat	LC	156 (121–35)	Carnivore	M	R – S
Prionodon pardicolor	Spotted linsang	LC	22 (1/21)	Carnivore	M	R – S
Rusa unicolor	Sambar	VU	27 (20/7)	Herbivore	L	R – S
Sus scrofa	Wild pig	LC	597 (201/396)	Omnivore	M-L	R – S
Trachypithecus phayrei	Phayre's leaf monkey	EN	1	Herbivore	M	R
Trachypithecus shortridgei	Shortridge's langur	EN	5	Herbivore	M	S
Ursus thibetanus	Asian black bear	VU	7 (6/1)	Omnivore	L	R – S
Viverra zibetha	Large Indian civet	LC	314 (230/84)	Carnivore	M	R – S
Viverricula indica	Small Indian civet	LC	1	Omnivore	M	R – S

Table S2. Occupancy (Ψ) and detectability (*p*) values in the three years of monitoring (1: 2016; 2: 2017; 3: 2018) for the selected species in the two areas (RYER: Rakhine and HWS: Sagaing). We reported median values with 95% Bayesian Credible Interval (BCI). Asterisks denote significant differences (no overlap between BCI) between occupancy and detectability values for the same species in the two areas during the same year.

Species	Area	Ψ1	Ψ2	Ψ 3	*p*1	*p*2	*p*3
Bos gaurus	RYER	0.12 (0.01–0.93)	0.12 (0.01–0.90)	0.06 (0.02–0.15)	0.01 (0.001–0.14)	0.01 (0.006–0.26)	0.08 (0.03–0.16)
	HWS	0.29 (0.04–0.96)	0.26 (0.02–0.85)	0.38 (0.09–0.88)	0.01 (0.002–0.09)	0.01 (0.008–0.28)	0.01 (0.003–0.09)
Catopuma temmincki	RYER	0.14 (0.04–0.58)	0.23 (0.10–0.59)	0.15 (0.08–0.29)	0.07 (0.02–0.17)	0.05 (0.02–0.11)	0.09 (0.04–0.16)
	HWS	0.54 (0.14–0.97)	0.30 (0.13–0.73)	0.26 (0.12–0.58)	0.01 (0.006–0.06)	0.02 (0.008–0.08)	0.04 (0.01–0.11)
Cuon alpinus	RYER	0.08 (0.02–0.59)	0.31 (0.08–0.88)	0.43 (0.09–0.89)	0.04 (0.004–0.16)	0.01 (0.004–0.06)	0.01 (0.003–0.06)
	HWS	0.25 (0.03–0.95)	0.33 (0.06–0.91)	0.23 (0.06–0.86)	0.01 (0.002–0.12)	0.01 (0.003–0.09)	0.07 (0.01–0.19)
Elephas maximus	RYER	0.10 (0.04–0.20)	0.12 (0.06–0.21)	0.10 (0.05–0.17)	0.10 (0.04 – 0.19)	0.08 (0.02–0.15)	0.14 (0.08–0.21)
	HWS	0.009 (0.003–0.41)	0.07 (0.02–0.33)	0.29 (0.08–0.77)	0.14 (0.05–0.27)	0.09 (0.02–0.20)	0.03 (0.007–0.12)
Hystrix brachyura	RYER	0.31 (0.22–0.44)	0.40 (0.31–0.51)	0.42 (0.32–0.53)	0.13 (0.09–0.17)	0.19 (0.15–0.23)	0.15 (0.11–0.19)
	HWS	0.29 (0.17–0.48)	0.36 (0.23–0.54)	0.23 (0.15–0.33)	0.10 (0.06–0.16)	0.10 (0.06–0.15)	0.20 (0.15–0.25)
Helarctos malayanus	RYER	0.23 (0.14–0.37)	0.36 (0.23–0.54)	0.31 (0.20–0.48)	0.11 (0.06–0.16)	0.09 (0.06–0.13)	0.10 (0.06–0.14)
	HWS	0.70 (0.33–0.98)	0.55 (0.20–0.93)	0.19 (0.08–0.58)	0.02 (0.01–0.06)	0.02 (0.01–0.06)	0.07 (0.02–0.17)
Homo sapiens	RYER	0.58 (0.48–0.67) *	0.55 (0.45–0.64)	0.60 (0.50–0.69)	0.25 (0.21–0.28)*	0.28 (0.24–0.31)*	0.31 (0.27–0.34)*
	HWS	0.21 (0.10–0.48) *	0.31 (0.18–0.57)	0.29 (0.16–0.53)	0.08 (0.03–0.15)*	0.08 (0.03–0.13)*	0.10 (0.04–0.15)*
Macaca leonina	RYER	0.30 (0.15–0.54)	0.38 (0.25–0.52)	0.39 (0.25–0.56)	0.04 (0.01–0.09)	0.08 (0.05–0.12)	0.06 (0.03–0.11)
	HWS	0.53 (0.22–0.98)	0.19 (0.08–0.49)	0.28 (0.12–0.64)	0.03 (0.01–0.09)	0.07 (0.02–0.15)	0.04 (0.01–0.10)
Muntiacus vaginalis	RYER	0.49 (0.37–0.63)*	0.61 (0.51–0.72) *	0.54 (0.43–0.65) *	0.12 (0.09–0.16) *	0.19 (0.16–0.22)	0.19 (0.15–0.22)*

	HWS	0.74 (0.64–0.83)*	0.86 (0.77–0.93) *	0.84 (0.76–0.91) *	0.23 (0.19–0.26) *	0.25 (0.22–0.28)	0.27 (0.24–0.30)*
Neofelis nebulosa	RYER	0.08 (0.03–0.14)*	0.07 (0.04–0.13) *	0.08 (0.04–0.14) *	0.12 (0.06–0.20)	0.01 (0.004–0.05)	0.01 (0.001–0.10)
	HWS	0.55 (0.23–0.95)*	0.40 (0.20–0.78) *	0.41 (0.23–0.74) *	0.03 (0.01–0.07)	0.05 (0.02–0.10)	0.07 (0.04–0.13)
Prionailurus bengalensis	RYER	0.58 (0.25–0.97)	0.38 (0.25–0.57)	0.49 (0.32–0.71)	0.03 (0.01–0.07)	0.08 (0.05–0.12)	0.04 (0.02–0.08)
	HWS	0.60 (0.22–0.98)	0.23 (0.10–0.60)	0.21 (0.07–0.62)	0.01 (0.008–0.05)	0.05 (0.01–0.12)	0.04 (0.01–0.13)
Paradoxurus hermaproditus	RYER	0.27 (0.1–-0.56)	0.53 (0.31–0.84)	0.57 (0.33–0.87)	0.07 (0.03–0.12)	0.05 (0.02–0.09)	0.04 (0.02–0.07)
	HWS	0.54 (0.42–0.68)	0.51 (0.33-0.76)	0.61 (0.35–0.90)	0.14 (0.10–0.18)	0.07 (0.04–0.11)	0.04 (0.02–0.07)
Pardofelis marmorata	RYER	0.009 (0.002–0.50)	0.12 (0.009–0.88)	0.58 (0.22–0.94)	0.07 (0.001–0.31)	0.01 (0.006–0.24)	0.01 (0.007–0.04)
	HWS	0.16 (0.06–0.56)	0.64 (0.28–0.94)	0.45 (0.16–0.88)	0.07 (0.01–0.16)	0.01 (0.009–0.05)	0.02 (0.009–0.07)
Sus scrofa	RYER	0.27 (0.16–0.48)	0.32 (0.20–0.53)	0.36 (0.21–0.63)	0.09 (0.04–0.14)	0.09 (0.06–0.14)	0.06 (0.03–0.11)
	HWS	0.33 (0.23–0.44)	0.56 (0.36–0.83)	0.66 (0.51–0.84)	0.16 (0.12–0.21)	0.09 (0.05-0.13)	0.12 (0.08–0.15)
Viverra zibetha	RYER	0.33 (0.2–0.45)	0.50 (0.34–0.68)	0.45 (0.30–0.65)	0.13 (0.09–0.17)	0.09 (0.06–0.13)	0.08 (0.05–0.11)
	HWS	0.36 (0.22–0.60)	0.29 (0.15–0.59)	0.37 (0.17–0.77)	0.08 (0.04–0.14)	0.07 (0.03–0.13)	0.04 (0.01–0.10)

Table S3. Colonization and persistence values between years (first and second (1) and second and third (2)) for the selected species in the two areas (RYER: Rakhine and HWS: Sagaing). We reported median values with 95% Bayesian Credible Interval (BCI). Asterisks denote persistence and colonization values significantly different during the same year in the same area (no overlap between BCI).

Species	Area	Colonization (1)	Persistence (1)	Colonization (2)	Persistence (2)
Bos gaurus	RYER	0.71 (0.04–0.99)	0.28 (0.07–0.95)	0.65 (0.04–0.98)	0.16 (0.05–0.85)
	HWS	0.63 (0.02–0.98)	0.30 (0.04–0.96)	0.73 (0.05–0.99)	0.43 (0.02–0.96)
Catopuma temmincki	RYER	0.76 (0.13–0.97)	0.39 (0.06–0.94)	0.52 (0.04–0.88)	0.30 (0.07–0.74)
	HWS	0.45 (0.13–0.93)	0.30 (0.05–0.89)	0.27 (0.12–0.72)	0.63 (0.21–0.97)
Cuon alpinus	RYER	0.81 (0.11–0.98)	0.65 (0.13–0.59)	0.73 (0.06–0.98)	0.36 (0.02–0.95)
	HWS	0.71 (0.02–0.99)	0.35 (0.01–0.96)	0.58 (0.03–0.98)	0.25 (0.04–0.95)
Elephas maximus	RYER	0.49 (0.06–0.82)	0.62 (0.25–0.96)	0.32 (0.02–0.70)	0.55 (0.24–0.88)
	HWS	0.95 (0.22–0.99)	0.47 (0.02–0.97)	0.91 (0.41–0.99)	0.34 (0.01–0.95)
Hystrix brachyura	RYER	0.50 (0.28–0.68)	0.64 (0.44–0.83)	0.45 (0.28–0.62)	0.57 (0.40–0.75)
	HWS	0.48 (0.12–0.73)	0.64 (0.34–0.95)	0.39 (0.06–0.67)	0.39 (0.21–0.62)
Helarctos malayanus	RYER	0.73 (0.47–0.89)	0.42 (0.17–0.77)	0.48 (0.14–0.73)	0.45 (0.23–0.77)
	HWS	0.21 (0.04–0.71)	0.62 (0.17–0.98)	0.46 (0.27–0.91)	0.18 (0.02–0.75)
Homo sapiens	RYER	0.31 (0.20–0.44)	0.65 (0.52–0.76) *	0.35 (0.23–0.47)	0.72 (0.59–0.83) *
	HWS	0.70 (0.21–0.91)	0.45 (0.14–0.91)	0.58 (0.12–0.85)	0.38 (0.13–0.84)
Macaca leonina	RYER	0.37 (0.03–0.72)	0.81 (0.43–0.99)	0.23 (0.02–0.54)	0.78 (0.47–0.99)
	HWS	0.26 (0.09–0.77)	0.25 (0.06–0.83)	0.60 (0.06–0.91)	0.58 (0.15–0.97)
Muntiacus vaginalis	RYER	0.36 (0.17–0.52)	0.81 (0.66–0.94) *	0.27 (0.14–0.41)	0.64 (0.50–0.77) *
	HWS	0.25 (0.15–0.36)	0.86 (0.76–0.95) *	0.10 (0.02–0.20)	0.88 (0.79–0.95) *
Neofelis nebulosa	RYER	0.09 (0.03–0.41)	0.89 (0.56–0.99) *	0.11 (0.04–0.49)	0.91 (0.61–0.99) *
	HWS	0.61 (0.02–0.95)	0.29 (0.04–0.83)	0.60 (0.10–0.88)	0.41 (0.12–0.89)

Prionailurus bengalensis	RYER	0.47 (0.01–0.84)	0.35 (0.14–0.64)	0.37 (0.05–0.63)	0.83 (0.50–0.99)
	HWS	0.26 (0.07–0.77)	0.28 (0.07–0.85)	0.44 (0.03–0.86)	0.49 (0.11–0.96)
Paradoxurushermaproditus	RYER	0.65 (0.23–0.86)	0.66 (0.27–0.97)	0.37 (0.03–0.70)	0.68 (0.33–0.98)
	HWS	0.28 (0.04–0.50)	0.68 (0.42–0.97)	0.40 (0.08–0.57)	0.73 (0.36–0.98)
Pardofelis marmorata	RYER	0.96 (0.21–0.99)	0.46 (0.02–0.97)	0.91 (0.50–0.99)	0.51 (0.02–0.97)
	HWS	0.85 (0.28–0.97)	0.60 (0.11–0.98)	0.35 (0.14–0.89)	0.44 (0.07–0.96)
Sus scrofa	RYER	0.82 (0.52–0.96)	0.21 (0.04–0.53)	0.63 (0.22–0.86)	0.42 (0.15–0.86)
	HWS	0.59 (0.38–0.76)	0.71 (0.40–0.98)	0.41 (0.08–0.66)	0.70 (0.46–0.95)
Viverra zibetha	RYER	0.52 (0.31–0.70)	0.74 (0.43–0.97)	0.27 (0.02–0.57)	0.65 (0.39–0.94)
	HWS	0.46 (0.06–0.68)	0.43 (0.17–0.88)	0.59 (0.12–0.87)	0.52 (0.15–0.96)
